# The English Version of the Health Profession Communication Collective Efficacy Scale (HPCCE Scale) by Capone and Petrillo, 2012

**DOI:** 10.3390/ejihpe10040075

**Published:** 2020-11-13

**Authors:** Vincenza Capone, Leda Marino, Anna Rosa Donizzetti

**Affiliations:** Department of Humanities, University of Naples Federico II, 80100 Naples, Italy; leda.marino@unina.it (L.M.); annarosa.donizzetti@unina.it (A.R.D.)

**Keywords:** communication efficacy, health organization, collective efficacy, doctors, Rasch model

## Abstract

Communication is a crucial component in all steps of the health care process. Therefore, it is important to have knowledge about the communication skills of the whole health organization. From the socio-cognitive perspective, collective efficacy beliefs are the main indicators of the capacity of functioning of the system. This work aimed to contribute to the validation of the English version of Health Profession Communication Collective Efficacy Scale (HPCCE scale) a self-report questionnaire measuring hospital doctors’ beliefs to succeed as a group to meet the needs of internal and external communication and of communication with patients, examining the structure, reliability and convergent validity. This study was a cross-sectional investigation conducted using snowball sampling. The participants were 287 doctors working at different hospitals in UK. Explorative factor analyses and Rasch analysis confirmed the one-factor solution. Results revealed high internal reliability. The HPCCE scale correlated positively with Social Self-Efficacy. The English version of HPCCE is a valid instrument to measure communication efficacy beliefs in hospital, involving different type of doctors. It can contribute to the implementation and evaluation of management interventions in a health organization aimed at its optimization.

## 1. Introduction

Over the last years, many works have dealt with doctor-patient communication, emphasizing the many advantages of effective communication for both interlocutors [1,2,3,4,5,6]. More specifically, a hospital doctor’s communication has a dual dimension. He/she is responsible both for the quality of communication with the patient, and together with other figures, for hospital communication. He/she has to coordinate individual work with that of others, is influenced by beliefs, the motivation and the quality of the performance of colleagues and other components of the structure [7].

The studies on health organization have noted that the presence of an effective communication strategy is one of the structural and organizational features to the success of programs for improving the success of the organization [8,9]. The achievement of optimal results in communication is a primary aim for the entire hospital, in which doctors are an essential part [10]. 

According to the model of human agency [11], from the perspective of social cognition, collective efficacy beliefs have a central role among the beliefs that most contribute to the smooth functioning of a working team and to the achievement of an effective performance [12]. Bandura [11] defines collective efficacy as the belief shared by a group about the abilities of group members to organize and implement a series of actions required to meet the organizational goal [13,14] referring to collective efficacy as the revelation of group-level characteristics and the result of group members’ interactions, and indicating that the characteristics displayed by the group are greater than the sum of individual-level characteristics.

On the subject, several studies attest the significant impact of efficacy beliefs on the functioning of the group and in achieving collective performance [11,15]. In the last years, tools for measuring collective efficacy have been developed for a variety of topics and populations [16], but few, however, have been deployed in health organization contexts [7,17].

An organization’s capacity of delivering better performance depends partly on the collective efficacy of its members. Usually, hospitals organize their staff into units or teams to maximize performance level through collective activities, such as team building. Hence, it is necessary to understand how collective efficacy influences health professionals’ performance in a unit. Depending on its collective efficacy, a unit would perform in a more synergistic manner than an individual health worker. In general, organizations with strong communication policies can enrich their patients’ health [18].

Hospitals involve a complex socio-technical health system [19], where communication failures influence the quality of an organization’s work and contribute to adverse clinical events and outcomes [20]. Health care professionals and institutions need to recognize the importance of communication in health care in order to thrive. In communication acts, health workers perceive not only their own capability but also capabilities of their colleagues and of the whole organization, and form the impression of how well the organization can produce the expected outcomes. In an economic perspective, the Stakeholder theory [21] pointed out that health organizations need to consider the importance of developing a comprehensive discourse with different stakeholders, and to incorporate their responses into health care scenarios. Freeman defines stakeholders as “any group or individual who can affect or is affected by the achievement of the organization’s objectives” [21] (p. 46). In the context of our study, stakeholders are patients, employees, users and external institutions. They expect a hospital to behave responsibly to relieve problems and to adequately present procedures and processes. Therefore, health organizations feel they must meet the expectation of the stakeholders to maintain a good reputation in an increasingly very complicated landscape, to maximize their performance and value generated, and to motivate employees and satisfy users [22]. In this perspective, communication plays a central role in serving the organizational process [23].

The communication collective efficacy of health organization refers to the beliefs of health workers, that their organization is able to communicate with different stakeholders. Conceptually, whereas collective efficacy involves members’ beliefs about the prospective capabilities of their group, the team performance is about the actual performance of a task. Despite this conceptual distinction, correlations between collective efficacy and performance have been extensively verified in the literature [24]. Hence, the communication collective efficacy of health organization could be a useful indicator of the performance of health workers.

On the subject, the Health Profession Communication Collective Efficacy Scale [7] was designed to assess collective beliefs of the physicians about the hospital’s capability to successfully manage, with the help of various health workers, problematic situations related to internal, external and with patients’ communication. The scale, consisting in 16 items, has a one-dimensional structure and excellent psychometric properties. It could be used in training aiming to enhance the individual and collective resources of health professionals. 

### 1.1. Communication in Health Organization

Effken [25] describes health care as a complex dynamic system in which people cooperate for patient care and are faced with numerous contingencies that often cannot be anticipated. Health care occurs in a variety of physical and organizational settings, but hospitals are the institutions that play one of the most important roles as they have an impact on the social, economic and environmental issues and also on health promotion [26].

Despite the division into wards and departments, a hospital gives an image of unity. This also applies to communication: the individual operators’ communication acts on hospital communication; hospital communication influences individual operators’ communication. Organizational communication is not only an exchange information between the two or more individuals or group in an organization, who created the common basis understanding and feelings [27]. When the members of the organization communicate with each other on an individual basis as well as in groups, they need to take into account the norms, values, standards and principles of the organizational culture [28]. It is not easy to define the characteristics of an effective communication at the organizational level. In general, “the communication system at an organization is both a reciprocal, dynamic process and a structural construct, determined by a set of internal and external factors, enabling horizontal, vertical, and diagonal information flow throughout the organization and also effectively and efficiently operating a number of communication categories with the aim to help individuals reach both their own and organizations’ communication goals, creating synergy among communicators” [29]. In the perspective of the stakeholder theory [21], stakeholder principles lead organizations to develop cooperative and trusting relationships with their major stakeholders, leading to higher levels of innovation, efficiency and value creation [30]. Based on these considerations, hospital communication fulfils some main functions, at different levels and with different stakeholders: (1) allowing the organization of the operational structure and the connection between the individual units; (2) improving the relationship between doctor and patient and accompany the therapeutic and diagnostic process; (3) improving the information relationship with social, family and territorial background [31]. So, each health care system has multiple forms of communication that administrators and staff must be trained to use properly and efficiently.

A hospital needs to manage internal communication between staff, to convey medical information about patients between departments quickly and with respect for privacy, but also to share the objectives of hospital corporation, retrain operators, and provide information service. 

Communication becomes a central element in the functioning of the hospital, both in the care organization and in the management of conflicts between health professionals and patients. Without communication, the quality of healthcare would be impaired. It falls within hospital corporation’s brief to promote among health care workers the importance of good communication and facilitate communication between staff and patient [32,33,34].

Health care corporations that do not update and fail to inform and to accommodate the patient adequately may create hostility in the patient before his/her meeting with the doctor [35].

Improving the quality of the relationship between the health care corporation and the territorial institutions, as well as with the citizen, which is the principal recipient of the services offered, is one of the objectives of external communication. In this regard, the “Health Services Charter” is the tool of communication par excellence. A hospital corporation is responsible for identifying and communicating information on how to access diagnostic and specialistic services. In the pre-reception phase, front-office staff must provide comprehensive information to users who need these services, including an information pack. Each hospital also has the task of implementing an Internet site containing key information on the structure and consistency with the Health on the Net Foundation specifications. Even listening to citizens and the management of complaints has taken, in recent years, an important role in the hospital’s organizational structure [36]. The hospital also has a central position in promoting health. As it is well explained in the “Vienna Recommendations on Health Promoting Hospitals” [37], hospitals, by reaching a broad sector of the population, can implement effective health promotion campaigns in the territory.

The lack of integration between the various members of the structure, inadequate technologies to facilitate communication, and disincentives, such as too long work shifts, are the main causes of communication problems in the hospital [38].

Clinicians, for example, put the blame for having little time for their patients and still having to cope with full waiting rooms on the lack of support by the health care organization: these two conditions make it impossible for effective evaluations and comprehensive answers to patients’ requests [39]. As Bandura [40] states, “now being the communication a tool essential in modern public organizations, both in the field of production processes and in the improvement of relations with citizens, but especially of the quality of services, it is necessary to strengthen the whole communication of health corporation”.

### 1.2. Collective Efficacy in Working Group and Organization

The socio-cognitive theory [11] pays a lot of attention both to personal and collective agency. It defines collective efficacy as “a group’s shared belief in its conjoint capabilities to attain their goals and accomplish desired tasks” [40] (p. 19). The members of a group not only must coordinate their individual work with the work of others, but are influenced by the beliefs, the motivation and the quality of the performance of their collaborators [11,41].

Perceived collective efficacy is interesting for several reasons. Firstly, group members’ perceptions on the group capacity to perform various tasks are a first indicator of the possible methods of undertaking the task itself. Moreover, collective efficacy is a valid and robust support to achieve group successes. The more the functioning of an organization is derived “from the capacity to access the skills of each and to concentrate their use in the service of common objectives, and the more the members have reason for trusting in the achievement of common goals, the more shared collective efficacy beliefs have a decisive role in supporting the commitment and trust” [42] (p. 10). The findings of an important meta-analysis showed that collective efficacy beliefs explain a good part of variance in the quality of group functioning [43]. In other words, collective efficacy is about the performance capability of the group as a whole.

From the socio-cognitive perspective, collective efficacy beliefs are the main indicators of the capacity of functioning of the system [41]. Stronger collective efficacy perceptions are related to higher aspirations of the group and to a motivational investment in its tasks, as well as to a stronger ability to control the setbacks, a higher morale, a greater resilience to stressors and a greater commitment [44]. Collective efficacy may influence the pursuit of success and how people manage their resources, projects, and strategies, as well as the efforts they make in group attempts and the vulnerability to discouragement [11,45,46].

People with high levels of collective efficacy orient their behavior towards the planning and use of shared resources and the willingness to persist, despite internal conflicts, changes in political or social concerns [47]. Collective efficacy is not simply the sum of individual efficacy beliefs: it is a group attribute resulting from the dynamics of interaction and cooperation. On the other hand, however, as Whyte [48] states, “inflated” perceptions of collective efficacy are possible antecedents to the failure in the implementation of specific tasks.

Zaccaro [49] defines collective efficacy as “a sense of collective competence shared among members when allocating, coordinating, and integrating their resources as a successful, concerted response to specific situational demands” [49] (p. 309).

As Karrasch [50] stresses, this definition encompasses several key elements: collective efficacy as a shared belief, the perceptions of competence in a collective’s coordination activities, the consideration of other members’ resources and the specificity of the situation, the behavior and the task of collective efficacy.

Collective efficacy motivates or demotivates individual behavior of the members of the group, influencing its goals and commitment to achieve them. Several studies show that efficacy beliefs contribute significantly to the levels of motivation and performance [51,52,53]. Karrasch [50] (p. 143) argued in this regard that “perceptions of collective efficacy influence what people choose to do as a group, their efforts and their power to stay together when the efforts of the group fail the objective”. This positive relationship between collective efficacy and team goals was confirmed by Prussia and Kinicki [53]: groups with high efficacy are more tied to their prefixed goals compared to groups with low efficacy. 

Probably one of the more recognized reasons for the growing interest in the construct is the positive link between collective efficacy and the aims achieved by the group. Many works have shown the strong correlation between collective efficacy and the implementation of good group performance: a high sense of collective efficacy, not illusory, but based on experience, determines the success of an organization and protects its solidity in times of difficulty and crisis [12,24]. Conversely, when high personal efficacy beliefs are associated with low collective efficacy beliefs, there is the basis for an increase of demotivation, disengagement and conflict that inevitably exacerbate or accelerate the decline of an organization [11].

Those with low levels of collective efficacy do not believe that the group is able to achieve its prefixed goals and this causes a state of apathy and indifference to it [54,55,56]: these perceptions directly affect the care and determination with which groups choose to pursue their own purposes. In a hospital setting, collective communication efficacy was correlated positively with well-being and quality of life [57] and negatively with burnout, and lack of professional fulfilment of health professionals (doctors and nurses) [58].

### 1.3. Aims and Hypothesis

The aim of the work is to contribute to the validation of the Health Profession Communication Collective Efficacy Scale (HPCCE scale) by Capone and Petrillo [7], a self-report questionnaire for doctor’ perceptions of collective efficacy in the communication of their hospital. We aimed to evaluate the validity of the English version of the scale by examining the structure, reliability and convergent validity.

We expect to confirm the mono-dimensional structure of the scale as found in the original study.

We hypothesize that the English version of HPCCE scale has a high internal reliability, similar to earlier findings in the Italian sample.

We hypothesize that our study confirms the convergent validity of the English version of the scale, correlating positively with a corresponding measure.

## 2. Materials and Methods 

### 2.1. The English Version of Health Profession Communication Collective Efficacy Scale

The English translated HPCCE was back translated to ensure translation equivalency. Psychometric testing of the HPCCE (English HPCCE) was then conducted. Two bilingual PhD researchers were involved in the translation process. One of the researchers who translated the original tool to English was a researcher in Social Psychology from a university in the UK. The other translator, who had been educated in Italy and had a PhD in Psychology, translated the English HPCCE back to Italian, without any discussion with the first translator. Subsequently, adjustments were conducted to ensure understandability, psychological equivalence, and the accuracy of the translation from Italian to English. The original and back-translated Italian versions did not differ appreciably as judged by the translators. 

The questionnaire was presented to a panel of experts to establish content (face) validity prior to conducting the study. This panel of experts consisted of three academic researchers, who have successfully validated measures of efficacy beliefs, and two UK hospital doctors.

Convenience sampling was then used to test the survey questionnaire to 10 doctors working in hospitals. They were asked to provide feedback on content for improvement. Feedback from respondents during this pilot test provided guidance for making necessary amendments on the questionnaire items [59].

### 2.2. Participants

This study was a cross-sectional investigation conducted using snowball sampling. The participants were 287 doctors working at different public hospitals in UK. Participants were initially recruited through direct contact with a researcher living and working in UK. Additional participants were recruited through snowball sampling guided by the social networking efforts of study participants. No compensation for participation was provided. Completion lasted 15 min. Doctors received an explanation of the study aims and reassurance of anonymity, confidentiality, and use of their responses solely for the purposes of the research. Subjects gave their informed consent.

The subjects were mostly men (M = 68.8% and F = 31.2%), with an age ranging from 27 to 69 years (mean = 48.65 years, SD = 10.02). Most of the doctors held an important position in the hospital: 60.0% of the sample were first-level managers, 20.0 % were second-level medical directors, 18.0% were responsible for the service, while 4.0% were specializing.

The length of service of the medical profession is 17.83 years on average (range = 1–40, SD = 9.73). The length of service in ward is instead 13.42 years (range = 1–37, SD = 9.71). The weekly working hours in the ward are on average 37.91 (range = 15–70, SD = 5.98).

### 2.3. Measures

The survey instrument was a self-report questionnaire that included, in addition to the HPCCE scale, a form for the collection of socio-demographic data and, to verify the construct validity, the scale of Perceived Social Self-Efficacy [60].

In its original version, the HPCCE scale consists of 16 items assessed on a five-point scale (1 = “not at all capable” to 5 = “completely capable”). It measures the beliefs of hospital doctors about their capacity of succeeding as a whole (physicians and other hospital professionals) to cope with different critical situations related to internal and external communication, and communication with patients.

The scale of Perceived Social Self-Efficacy notes individuals’ beliefs relating to their capacity of fitting easily, feeling at ease and playing a proactive role in social, sometimes new, situations. It consists of 15 items; for each item, respondents rated to what extent they considered to be capable of managing social relations on a five-point scale (1 = “not at all capable”, 5 = “very capable”).

### 2.4. Procedure

The first step, in order to validate the HPCCE scale, was an exploratory analysis of data collected. To this end, using the SPSS 21.1 software [61], normality was tested through skewness and kurtosis, and the number of latent dimensions was determined by Factorial Analysis (method of principal axis factoring and Oblimin rotation of the factors) and through Scree test; finally, the internal consistency of the scale was measured with Cronbach’s Alpha.

Subsequently, the Rasch model was used to confirm the mono-dimensionality of the scale and to obtain a more accurate analysis of the items. To this end, using the software RUMM 2030 [62], it was possible to calculate the overall fit of the model—which provides a summary measure of adaptation of the model to the data—and the indices of adaptation of the items, with particular reference to their significance in terms of Chi-square (items possessing values of *p* > 0.05 adhere consistently to the latent dimension), to residuals (normal values are considered between −2 and +2) and to the thresholds (Andrich [62] speaks about thresholds as he assumed that between a category and the following one that there is a threshold, a kind of border which is a parameter that "qualifies" the item position).

## 3. Results

### 3.1. Exploratory Analysis

Skewness and kurtosis of the 16 items of the scale have shown that all the items are near the normal curve. Therefore, Factorial Analysis was conducted on all the items, from which two factors emerged with eigenvalue > 1: the first explains the 57.85% of the total variance (eigenvalue = 13.69), while the second factor explains 2.97% of the total variance (eigenvalue = 1.06). The reading of the Scree test has suggested the interpretation of a single dimension. Therefore, the analysis was again carried out with the extraction of a single factor that explains the 57.72% of the total variance. The saturation of the items ranges from 0.82 to 0.67. The Cronbach’s Alpha coefficient was very high, equal to 0.97, as well as from the analysis of item-total correlations which did not yield the required elimination of any item (Table 1).

### 3.2. Mono-Dimensionality Tested through the Rasch Model

An analysis conducted on the responses given by participants to the 16 items of the HPCCE scale founded that the Chi-square—index for the assessment of levels of total matching—cannot be considered satisfactory (χ^2^ = 129,679 (92), *p* = 0.006), although the index of separation, sensitive to the discriminatory power of the categories of response is very good (excellent: 0.96). Therefore, the requirements outlined above were more deeply analyzed and assessed: first, the thresholds are all ordered, that is, there is a correct choice of the number of modes of response in order to detect all the different positions of the participants contacted; second, the analysis of the items showed no items had significance levels below 0.05 or residual non-acceptable. So, the English version of the scale consisted of 16 items with five modes of response, has a probability of Chi-square > 0.05 (χ^2^ = 68,538 (64), *p* = 0.33) and excellent separation index (0.95) (Table 2).

The test of item-person adaptation indicates that there was a good consistency of patterns of responses of the participants and the items (Table 3). The standardized average of residuals’ fit of the items was −0.06 and the relative standard deviation was 1.13, the standardized average of residuals’ fit of persons was −0.46 and the relative standard deviation is 1.77.

### 3.3. Construct Validity 

The construct validity of the English HPCCE scale has been tested through an analysis of the bivariate correlations between it and the scale of Perceived Social Self-Efficacy (Pastorelli and Picconi, 2001), as in the original validation study. The two scales were positively and significantly correlated (r = 0.37, *p* < 0.001).

The validation procedures show that the HPCCE scale measures hospital doctors’ beliefs to succeed as a group (physicians and other professionals) to meet the needs of internal and external communication and of communication with patients (Appendix A).

### 3.4. Descriptive Data for the Whole Sample 

As for the variables considered, we found the following trends. Doctors have low levels of perceived collective efficacy in communication in the hospital (2.30, SD = 0.78). They have, however, higher values of social perceived efficacy (3.20, SD = 0.74): they considered themselves, in fact, quite able to fit easily in a group and play a proactive role in social situations.

### 3.5. Differences between Groups According to Length of Service 

The participants were divided into three groups according to the working years of the medical profession: (a) young doctors (from 1–10 years), (b) doctors in career (11–21 years), (c) medical experts (22–37 years).

The univariate analysis of variance (Table 4) and post hoc Tukey test showed that the doctors in career and young doctors have levels of collective efficacy in communication in the hospital higher than medical experts (doctor in career = 2.42, young doctors = 2.21, medical experts = 2.06). There are significant differences between the groups, however, with regard to perceptions of social self-efficacy.

## 4. Discussion

The shared beliefs of people in their collective power to attain desired goals are a key ingredient of collective action. The results achieved by a group are, in fact, not only the product of knowledge and skills shared by its members, but also of interactive and synergic dynamics of their transactions [63]. Bandura [11] introduced the concept of collective efficacy as an extension of the social cognitive theory at the group level. A high and widespread feeling of collective efficacy, which results from the tests actually passed and the successes achieved, is a driving force allowing the best use of social system resources [44]. In the context of interdependence, “as many organizational contexts, success (also) depends on beliefs of collective efficacy, namely the practices of acting together and the beliefs that sustain these practices” [64] (p. 16). The sense of efficacy also plays a key role in human functioning not only directly but also through the influence it exerts on other important personal determinants, such as objectives and aspirations, expectations of performance, emotional inclinations, perceptions of obstacles and social opportunities. Many of the results that people reach are available only through efforts that are interdependent: the effectiveness of a health care organization depends on the multidisciplinary collaboration of a working team, on its ability to communicate, integrate, share and collaborate. The hospital has to ensure successful communication: internal, external and with patients. Effective communication, both intrahospital and interhospital, is important for providers to protect their patients, save on costs, and increase day-to-day operating efficiency [1]. For an optimal functioning of the organization, increasing importance has not only examined the perceived quality of service to users, but also analyzed the perceived communication capacities of the system by the staff.

The scale presented in this work aims to measure the hospital professionals’ beliefs to face, as a group, specific situations they may encounter at the hospital with reference to the communication between the various components of the hospital, between health professionals and patients, and between the hospital and the background. The psychometric characteristics of the English HPCCE scale were similar to those of the original version. The instrument has shown good properties, and the use of Rasch model has enabled not only its streamlining through a more careful analysis of the items [65], but also to ensure that perceptions about hospital communication beliefs are not differentiated in different dimensions, as aspects related to internal, external communication and communication with patients are part of a single dimension of collective communication efficacy.

The results of correlational analyses highlighted the goodness of the instrument in identifying the construct of our interest. The positive correlation found between the scale of perceived HPCCE and the scale of Perceived Social Self-efficacy means that there is a degree of interdependence between the constructs, as both reported to efficacy perceptions in areas brought together by their common relational feature; but considering the values of the correlation, this is not, such as, to call into question the relative autonomy of the constructs. This result lays an emphasis on the nature of collective efficacy as a socially shared perception and an emergent property of the social system. It does not coincide with the simple sum of perceptions of personal efficacy [49], even when the areas of functioning are the same. This finding contributes to the theoretical debate on the relative autonomy of these two constructs [16,66]. However, in future studies, it would be appropriate to use an organizational efficacy scale for convergent validity.

The descriptive analysis results are in line with the literature, which generally shows the tendency of individuals belonging to different spatial and organizational backgrounds to express collective efficacy beliefs lower than self-efficacy assessments [67]. This is understandable according to an optimistic bias in the assessment of self-reported features, involving, more directly, aspects of their identity, while assessments related to aspects of a collective entity, which inevitably are influenced by the social shared image of the organization, are those with whom they can identify themselves in a different individual way, including mutual images that tend to develop between the various components.

The assessment of doctors’ collective communication efficacy refers, in fact, also to their assessment of communicative efficacy of departmental colleagues, nurses, and managers of the structure, and to their assessment of the capacity of integration between these different competences. The English version of HPCCE results as a useful scale to retain the differences between subgroups of doctors. In this sense, length of service within the hospital was particularly significant, which has highlighted a gap between medical experts, with more than 20 years of work, who expressed greater confidence in communicative collective efficacy, and other groups. Probably, their perceptions are slightly, but significantly less negative for different reasons, and different interpretations about it make clearly understood the choices made in attributing the labels to the subgroups of doctors. One can infer that for those working less time in hospital, it was not yet possible to fully assimilate the organizational culture and establish the continuity of practices, routines and decision-making processes in co-constructed and collaborative ways, which characterize working groups at high interdependence; neither were they able to have adequate positive confirmations of the practices shared that were recently undertaken, to know the consequences in the medium and long term of choices made in a team and with a shared responsibility. For young doctors, therefore, less pessimism towards the efficacy of communication is related to a still imperfect knowledge of organizational mechanisms that, combined with an ideal charge toward the corporate mission, leads them to develop a minor criticism compared to older doctors. Their adjustment around values closest to mean scoring makes them closer to doctors in career, who are more internal to organizational processes and hold management positions or are closest to achieving goals of success, so they are also more motivated to contribute and enhance the development of a positive atmosphere and a cooperation in working groups.

We must also consider the possibility of a larger impact on perceptions of older doctors with malpractice experience in the specific contexts of belonging, and of recent changes in public health organizational structures, which have strongly marked the Italian health system: these elements, taken together, may have contributed to their increased pessimism and to a lesser identification with the hospital.

## 5. Limitation

The cross-sectional design of our study limits the interpretation of our findings and did not allow us to infer causal relationships. Watson et al. [68] found that collective efficacy was relatively stable over time: it will be necessary to verify the stability of the measure with a test-retest reliability. We also recommend that future researchers consider a longitudinal design that would provide greater clarification regarding the relationships between variables. Future studies may also benefit from gathering collateral information from employing organizations in order to establish the convergent validity of participants’ efficacy beliefs and to enhance the interpretability of our results [69]. All participants worked in public hospitals. Research evidence suggests that globally, health care personnel in public hospitals work under appalling conditions [70]. Future studies should take into account the differences between public and private structures. Finally, we were limited by our convenient and small sample size. The sample was not balanced by gender. Even if in 2019, there were approximately 162.1 thousand registered doctors in the United Kingdom who were male, compared to 139 thousand females [71], future studies should investigate the specific contribution of demographic variables.

Another limitation concerns the variety of the number of hospitals participating in the study. This latter limit did not allow to use organization as a unit of analysis; considering that we took into account efficacy at the collective level, this might be expected. Thus, future studies should try to collect data in a higher number of hospitals and track participants’ health organizational affiliation in an effort to identify and reduce possible bias in recruitment. The self-report instruments have the potential for issues of social desirability bias. Although we need to consider this limitation, it is reasonable to think that our data was not highly influenced by this bias because anonymity was guaranteed in data collection [72].

Finally, although the original version of the scale was developed thinking of the concertation between all the components of the hospital, our work presented the results of the validation for doctors. A validation study of the HPCCE scale with nurses and other professionals, such as technical and administrative profiles, equally involved in communicative practice, would be desirable.

## 6. Conclusions

We can conclude that findings from the current study provide evidence for the factorial validity and reliability of the HPCCE scale. This instrument covers a methodological and instrumental gap in the existing literature and responds to a very specific need in health communication. Based on the good psychometric properties of the scale, we recommend a wide use of it in health communication research. Therefore, it could be usefully employed with different purposes of intervention. The scale can be best applied, in fact, in the empirical study of factors that affect communication in the hospital. Hoping for, more generally, an increasing focus on communication by health professionals, this tool can also be used in training aimed at enhancing communication skills directed to various professions involved in prevention, treatment and rehabilitation, and to develop a sense of membership and collaboration within a hospital community. Reasons for measuring the quality of care include identifying potential areas for improvement. The HPCCE scale could be used as a tool in local quality improvement initiatives as well as in national health care strategies. Hospital managers should recognize the characteristics of a working group (for example: leadership style, efficacy of a leader, atmosphere of a team, collective efficacy).

The English version of the scale can be applied optimally to assess potential organizational problems prior to conducting major interventions; investigate dynamic problems in communication; target interventions designed to enhance perception of efficacy beliefs, and incorporate evaluation of communication collective efficacy as part of regular employee assessments. In future developments, we expect to verify the stability of the instrument in different populations implicated in hospital practice. The scale should also be tested and validated in languages other than Italian and English for usage across cultures.

## Figures and Tables

**Table 1:** Exploratory Factorial Analysis, item-total correlation and Cronbach’s Alpha if item deleted.

	Factors’ Loading	Item-Total Correlation	Cronbach’s Alpha If Item Deleted
Item 12	0.82	0.81	0.97
Item 4	0.82	0.80	0.97
Item 2	0.81	0.79	0.97
Item 10	0.80	0.79	0.97
Item 6	0.80	0.79	0.97
Item16	0.80	0.79	0.97
Item15	0.79	0.78	0.97
Item 3	0.79	0.77	0.97
Item11	0.78	0.77	0.97
Item 8	0.77	0.76	0.97
Item 9	0.77	0.76	0.97
Item 13	0.77	0.75	0.97
Item 7	0.76	0.75	0.97
Item 5	0.75	0.74	0.97
Item 14	0.75	0.74	0.97
Item 1	0.75	0.74	0.97

**Table 2:** Items’ order of Health Profession Communication Collective Efficacy Scale (HPCCE) according to scores in logit (Location) and its residues (FitResid), Chi-square (ChiSq), grades of liberty (DF) and probability of Chi-square (Prob).

Item	Location	FitResid	ChiSq	DF	Prob
8	–0.506	–0.39	4.624	4	0.33
11	–0.495	–0.30	7.774	4	0.10
5	–0.266	1.82	2.710	4	0.61
13	–0.238	1.72	9.017	4	0.06
16	–0.234	–1.39	8.037	4	0.09
6	–0.202	–0.47	0.576	4	0.97
9	–0.148	0.10	1.598	4	0.81
15	–0.097	–0.59	6.898	4	0.14
2	–0.065	–0.17	0.184	4	1.00
3	0.101	0.17	2.134	4	0.71
4	0.132	–0.40	5.038	4	0.28
1	0.208	1.43	1.442	4	0.84
7	0.284	0.54	1.874	4	0.76
12	0.302	1.05	7.977	4	0.09
14	0.323	–1.65	6.663	4	0.15
10	0.903	–1.83	1.993	4	0.74

**Table 3:** The test of item-person.

	ITEMS		PERSONS	
	Location	Fit Residual	Location	Fit Residual
Mean	0.00	–0.06	–1.58	–0.46
Standard dev	0.36	1.13	1.94	1.77

**Table 4:** Analysis of variance. Differences between groups according to working years of the medical profession.

	Young Doctors N = 76	Doctors in Career N = 97	Medical Experts N = 101	F(df)	*p*
HPCC	2.28 ^a^	2.43 ^a^	2.06 ^b^	6.325 _(2,281)_	0.02
Social self-efficacy	3.14	3.16	3.24	6.845 _(2,140)_	0.63

^a^ and ^b^ show graphically the results of the Tukey test.

## Appendix A

Below, you will find a series of statements concerning the Hospital where you work (along with all other coworkers). Mark, for each proposed statement, the score that comes closest to its location.

**Table A1:** The English Version of the Health Profession Communication Collective Efficacy Scale.

➀	➁	➂	➃	➄
**Nothing**	**Little**	**Enough**	**Very**	**Completely**

In which instance is Assess of the Hospital where you work capable of achieving as specified below? Remember that Hospital refers to you, along with all other hospital workers: thus “My Hospital” means “I, along with all other operators”.

1. My hospital is able to provide contacts between the company and users by implementing effective health promotion campaigns on the territory.
2. My hospital can achieve the goal of good communication by providing a Service Charter that is comprehendible and accessible.
3. My hospital is able to facilitate communication with other public and social services operating in the area.
4. My hospital is able to provide opportunities to qualified medical staff to update on all patient communications.
5. My hospital knows how to foster information to various operational units, in the exchange of patient information quickly, while keeping privacy in respect.
6. The hospital where I work is able to face difficult issues, confronting operators to develop viable solutions.
7. The hospital where I work is able to demonstrate, with clear communication and motivation, strengths, and those that could be improved through joint work.
8. My hospital is capable of sending out a positive image of itself.
9. My hospital is able to achieve the goal of ensuring that optimum communication operators in the front office are knowledgeable and helpful.
10. The various health workers of the hospital where I work, know that working together can ensure optimal communication with the patient.
11. The hospital where I work is able to meet the needs and gather any reports or complaints by patients.
12. My hospital is able to foster communication between myself and the other operators.
13. The hospital where I work is able to ensure adequate communication between health professionals and administration.
14. My hospital is able to ascertain the strengths and weaknesses of the company by doing surveys among operators and analyzing the information provided.
15. My hospital is able to ensure that inner hospital communication ensures adequate organization with the patients, such as avoiding overcrowded waiting rooms.
16. My hospital is able to provide adequate space to allow communication with patients in quiet and privacy.
